# Will Chemical Exposomics Be Ready for a Human Exposome Project?

**DOI:** 10.1021/acs.est.6c03509

**Published:** 2026-04-20

**Authors:** Jonathan W. Martin

**Affiliations:** † Science for Life Laboratory, Department of Environmental Science, 7675 Stockholm University, 114 18 Stockholm, Sweden; ‡ National Facility for Exposomics, Science for Life Laboratory, Stockholm University, 171 77 Solna, Sweden

**Keywords:** exposome, environmental pollutants, Human Exposome Project

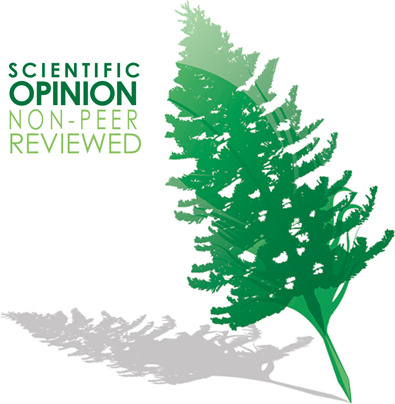

## Graphical abstract

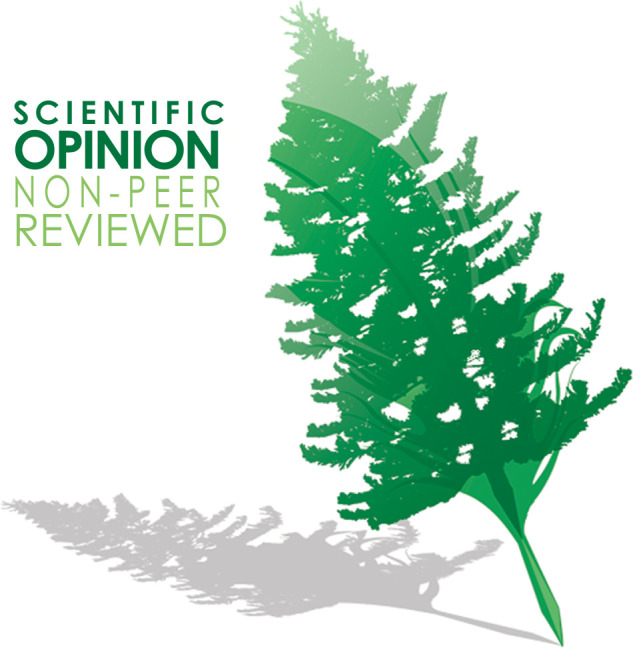

In an analysis of the major scientific themes appearing in *Environmental Science & Technology* (*ES&T*) over its first 60 years, the exposome did not exactly stand out.^1^ To be fair, the term has only existed since 2005 ^2^ and did not even appear in *ES&T* until 2011 when, then Editor-in-Chief, Jerold Schnoor enthusiastically embraced the topic and welcomed submissions.^3^ Noting that the exposome was a measure of all exposures that an individual accrues in a lifetime, including to many environmental pollutants that had long been a focus of the journal, he warned that it will be “exceedingly difficult to measure due to extreme variability in space and time of a single individual’s exposure”. Notwithstanding his optimism that the scale of discoveries in exposomics could dramatically improve health, he was right that this was going to be hard.

Today, *ES&T* is a primary target journal for research on the exposome, and a special issue on The Exposome and Human Health was recently compiled.^4^ As new innovative approaches, tools, and resources emerge, it is hard not to be optimistic that this field will transform the health sciences: I am. Nevertheless, exposomics remains a young field that is still dominated by many forward-looking expert opinions and editorials setting out the ambitions, scope, definitions, and frameworks for future studies. In contrast, recent headlines suggest that exposome research has come of age,^5^ implying a level of maturity that is ready to be unleashed, and international discussions for a Human Exposome Project involving millions of participants are now being amplified in popular media.^6^ Expectations are sky-high that such an endeavor will complement the wealth of information already gained from the Human Genome Project and postgenomic era, finally leading us to healthier populations and more precise medical treatments for sick patients. This is a grand challenge from which we cannot afford to back down, but like a team of climbers preparing for an uncharted summit, trustworthy equipment, coordinated teamwork, and timing of the ascent will be critical.

As one of a handful of researchers striving to develop sensitive, comprehensive, reproducible, and high-throughput methods to measure the chemical component of the exposome (chemical exposomics), I wonder how soon we can realistically be ready for a Human Exposome Project. When the timing is right—politics aside, the funding is not yet in place—I would not want imperfect methods to hold the project back. Here I reflect on the necessity of chemical exposomics in large-scale exposome research, its origins, the progress to date, and remaining challenges.

Environmental and health scientists are now forming research synergies to identify the major drivers of disease, motivated by a common understanding that an individual’s environmental exposures are as important as genetics in determining one’s risk of chronic disease and premature mortality.^7,8^ From my view, the missing piece in health research is no longer just “environment” in the abstract and hand-wavy sense; it is the chemical exposome, the complex mix of chemicals and particle-associated stressors that seep into our body’s cells daily through air, water, food, the microbiome, and commercial products. Scientific links between environmental chemical exposures and cancer are already 250 years old,^9^ and I can only surmise that Christopher Wild’s own research on aflatoxin or nitrosamine exposure^10^ and genotoxicity^11,12^ influenced his initial articulation of the exposome concept.^2^ Air pollution causes millions of premature deaths globally every year, and the already staggering burden of disease from chemical pollution remains underestimated due to data gaps for thousands of chemicals in global commerce.^13,14^ Links between environmental exposures and disease continue to pile up; e.g., PFAS and immune dysfunction,^15^ air pollution and dementia,^16^ and bisphenols and neurodevelopmental deficits.^17^ However, the pace of discoveries lags far behind ramping chemical production and the release of new pollutants to the environment.^18^ While I certainly acknowledge the importance of physical and psychosocial determinants of health, these nonchemical stressors may also be shown to be propagated by chemical signals that impact health through biomolecular interactions; as with chronic stress triggering cortisol production.^19^ In short, a human exposome project without chemical exposomics would be like mapping the night sky while ignoring the stars.

Chemical exposomics is powered by a set of emerging technologies, workflows, and data science tools that together allow for the complex chemical component of the exposome to be detected and characterized. When applied to blood, tissues, or urine, the same tools can simultaneously profile endogenous metabolite patterns, opening great opportunities to detect an associated biological response.^20^ Largely propelled by advances in high-resolution mass spectrometry (HRMS), current methods for small molecule profiling were only a vision a decade ago. We can now screen quantitatively for hundreds of priority target analytes and metabolites in a few drops of blood from one individual, while simultaneously scanning tens of thousands of nontarget signals to discover novel or unanticipated exposures.^21^ Ten years ago, to analyze the few hundred priority substances monitored by the U.S. Centers for Disease Control and Prevention in one person’s blood sample, one would have needed to send separate aliquots to dozens of specialized laboratories, and that would have required 250–300 mL of plasma.^22^ Not only was molecular discovery not integrated with routine biomonitoring, the needed blood volumes and high costs meant that comprehensive target analysis was reserved for pooled samples or that each chemical class was analyzed in blood of different individuals.

Through chemical exposomics, exposure science is now “personal”. Only fractions of a milliliter of blood are needed for multiclass target analysis, and combined nontarget/target methods allow for interindividual variability and longitudinal dynamics to be revealed for thousands of chemical signals.^23–27^ Molecular discoveries and chemical identifications are aided by increasingly large and public spectral databases and chemical libraries.^28,29^ Machine learning is already helping to flag hazardous chemicals among the unidentified nontarget features.^30,31^ Wearable samplers can track personal airborne exposures,^24^ including in passive devices that absorb chemicals like tiny sponges.^32^ Archived newborn blood spots can reveal complex prenatal exposures years later,^33^ and personal microsamplers allow research subjects to safely and painlessly draw their own blood samples at any place and any time.^34^ What would surely have sounded like science fiction a decade ago, mapping and recording the complexity and dynamics of an individual’s lifetime chemical fingerprint, is now technically feasible. We know that each person’s exposure cannot be adequately represented by a population average and chemicals do not politely take turns affecting biology one at a time, so advances in chemical exposomics are a game changer.

Truthfully though, chemical exposomics is not yet plug and play. With some effort and (admittedly) expensive infrastructure, partial chemical exposomes have been measured by nontarget methods at the cohort scale for hundreds of participants using either GC- or LC-HRMS, but so far not by both for the same individuals. Throughput and method harmonization remain barriers to larger studies. Moreover, no individual’s chemical exposome has been recorded for more than two years, and at intervals of only 3–6 months.^23^ Measuring the chemical exposome of any one individual over the course of a life remains a distant ambition. Among the tens of thousands of chemical signals that can be detected in human and environmental samples, most remain unidentified, and debates are ongoing about the significance of this “molecular dark matter”.^35^ Without clear chemical identities, health intervention and regulation must wait. Reproducibility is another hurdle. Different laboratories, instruments, and pipelines can yield different molecular profiles for the same samples. Standardization efforts are underway, but the field is still far from the crisp comparability that genomics eventually achieved. Then there are the obvious statistical and computational challenges for large studies, and even small studies will require advanced bioinformatics or machine learning and artificial intelligence. The high likelihood of false positives looms large in high-dimensional chemical exposome data sets.

For chemical exposomics to eventually complement genomics, it needs to aim for similar levels of reliability, throughput, and cost. The evolution of genomic technologies is therefore useful context, and that story began with a 25-year head start. Scientists launched the Human Genome Project in 1990 despite extremely high costs and low throughput by the best available technologies, largely Sanger sequencing. Completed by 2003 through intense international cooperation,^36^ it was propelled by steadily improving technologies and major investments in organized infrastructures. It was not until the first draft of the human genome sequence was imminent for publication that genomics was said to have “come of age”, and that was only after exponentially increased rates of sequencing and public data deposition were being recorded.^37^ With a final price tag in the range of $1–3 billion to sequence a reference human genome, it was clear that full genomic sequencing was still not applicable to individuals. Precision medicine therefore awaited further investment and innovation, culminating in next-generation high-throughput sequencing technologies by the mid-2000s.^38^ Today, a human genome can be sequenced in days to weeks for less than $1000,^39^ enabling routine applications in precision health and medicine.

While a philosophical, methodological, and technological revolution is ongoing in chemical exposomics, with genomics as context the methods have had just too little time and investment to reach anything equivalent to maturity or next-generation status. Realistically, chemical exposomics is still in its “Sanger sequencing” era, battling low throughput, high costs, and the lack of off-the-shelf commercial kits and still building the national infrastructures that will be needed to handle the demands of any Human Exposome Project. However, just as in the Human Genome Project, we will not have the luxury to wait for the perfect technologies and methods before embarking on large-scale international studies, and any technologies will be certain to evolve by leaps and bounds throughout any future study’s lifetime. Instead of a focus on when specific methods or technologies will be ready to go, the focus should be on data quality and data transparency, with the aim of ensuring future data comparability from a range of evolving methods. International dialogue toward the adoption of common data quality and reporting standards and the development of reference solutions and standard reference materials should be high priorities in the short term.

The trajectory for collaborative action looks excellent as national chemical exposomics infrastructures are coming online across Europe and the United States, increasingly interconnected by coordinating bodies (e.g., NEXUS and EIRENE).^40,41^ For chemical exposomics specifically, an international effort involving 20 laboratories is now underway to generate an online atlas of the chemical exposome in human biofluids.^42^ New international scientific constellations have developed,^43^ and the *Washington Declaration* now gives the field a road map to establish the foundations of a Human Exposome Initiative.^44^ Such an initiative was recently proposed to the European Parliament that would involve 10 million participants;^45^ therefore, time is of the essence, and the need to work collaboratively is obvious.

Chemical exposomics is not a silver bullet and will not untangle every environmental cause of disease; however, it is a measurement technology we have long lacked, and measurement can change everything. Once the field emerges from its necessary but disjointed phase of early innovation, I expect the trajectory to look like that of genomics, with consolidation, method maturation, and strong commercial partnerships to follow. If the field can resist hype, invest in rigor, and stay grounded in accurately revealing exposure, chemical exposomics stands to fundamentally reshape exposure science, public health, and even medicine.^46^

In closing this Viewpoint in honor of *ES&T* at 60, I hope and fully expect the journal to play a leading role in communicating new science on the exposome for years to come. An academically diverse range of editors, editorial board members, and reviewers must continue to be engaged to maximize quality and impact in this burgeoning transdisciplinary field.

## References

[1] Lowry G. V., Ren Z. J., Alvarez P. J. J., Hollender J., Wang S., Mills M., Zimmerman J. B. (2026). Global
Impact of 60 Years of *ES &T*. Environ. Sci. Technol..

[2] Wild C. P. (2005). Complementing
the Genome with an “Exposome”: The Outstanding Challenge
of Environmental Exposure Measurement in Molecular Epidemiology. Cancer Epidemiol., Biomarkers Prev..

[3] Schnoor J. L. (2011). Obesogens,
the Exposome, and ES&T. Environ. Sci. Technol..

[4] Gago-Ferrero P., Cousins I., Ghassabian A., Lamoree M., Schlenk D., Toms L.-M., Wang B., Zimmerman J. (2025). The Exposome
and Human Health. Environ. Sci. Technol..

[5] Beans C. (2025). Exposome Research
Comes of Age. Proc. Natl. Acad. Sci. U.S.A..

[6] Carr, G. The Human Exposome Project Will Map How Environmental Factors Shape Health. Economist, February 18, 2026. https://www.economist.com/science-and-technology/2026/02/18/the-human-exposome-project-will-map-how-environmental-factors-shape-health (accessed 2026-02-25).

[7] Rappaport S.
M. (2016). Genetic
Factors Are Not the Major Causes of Chronic Diseases. PLoS One.

[8] Argentieri M. A., Amin N., Nevado-Holgado A. J., Sproviero W., Collister J. A., Keestra S. M., Kuilman M. M., Ginos B. N. R., Ghanbari M., Doherty A., Hunter D. J., Alvergne A., van Duijn C. M. (2025). Integrating the Environmental and
Genetic Architectures
of Aging and Mortality. Nat. Med..

[9] Azike J. E. (2009). A Review
of the History, Epidemiology and Treatment of Squamous Cell Carcinoma
of the Scrotum. Rare Tumors.

[10] Wild C. P., Umbenhauer D., Chapot B., Montesano R. (1986). Monitoring
of Individual Human Exposure to Aflatoxins (AF) and N-nitrosamines
(NNO) by Immunoassays. J. of Cellular Biochemistry.

[11] Van
Benthem J., Wild C. P., Vermeulen E., Winterwerp H. H., Den Engelse L., Scherer E. (1988). Immunocytochemical
Localization of DNA Adducts in Rat Tissues Following Treatment with
N-Nitrosomethylbenzylamine. IARC Sci. Publ.

[12] Wild C. P., Garner R. C., Montesano R., Tursi F. (1986). Aflatoxin B_1_ Binding to Plasma Albumin and Liver DNA upon
Chronic Administration
to Rats. Carcinogenesis.

[13] Fuller R., Landrigan P. J., Balakrishnan K., Bathan G., Bose-O’Reilly S., Brauer M., Caravanos J., Chiles T., Cohen A., Corra L., Cropper M., Ferraro G., Hanna J., Hanrahan D., Hu H., Hunter D., Janata G., Kupka R., Lanphear B., Lichtveld M., Martin K., Mustapha A., Sanchez-Triana E., Sandilya K., Schaefli L., Shaw J., Seddon J., Suk W., Téllez-Rojo M. M., Yan C. (2022). Pollution and Health:
A Progress Update. Lancet Planetary Health.

[14] Landrigan P. J., Fuller R., Acosta N. J. R., Adeyi O., Arnold R., Basu N., Baldé A. B., Bertollini R., Bose-O’Reilly S., Boufford J. I., Breysse P. N., Chiles T., Mahidol C., Coll-Seck A. M., Cropper M. L., Fobil J., Fuster V., Greenstone M., Haines A., Hanrahan D., Hunter D., Khare M., Krupnick A., Lanphear B., Lohani B., Martin K., Mathiasen K. V., McTeer M. A., Murray C. J. L., Ndahimananjara J. D., Perera F., Potočnik J., Preker A. S., Ramesh J., Rockström J., Salinas C., Samson L. D., Sandilya K., Sly P. D., Smith K. R., Steiner A., Stewart R. B., Suk W. A., van Schayck O. C. P., Yadama G. N., Yumkella K., Zhong M. (2018). The Lancet Commission on Pollution and Health. Lancet.

[15] Bline A. P., DeWitt J. C., Kwiatkowski C. F., Pelch K. E., Reade A., Varshavsky J. R. (2024). Public
Health Risks of PFAS-Related Immunotoxicity
Are Real. Curr. Envir Health Rpt.

[16] Best
Rogowski C. B., Bredell C., Shi Y., Tien-Smith A., Szybka M., Fung K. W., Hong L., Phillips V., Jovanovic Andersen Z., Sharp S. J., Woodcock J., Brayne C., Navaratnam A., Khreis H. (2025). Long-Term Air Pollution Exposure
and Incident Dementia: A Systematic Review and Meta-Analysis. Lancet Planetary Health.

[17] Zhang J., Yuan M., Liu Y., Zhong X., Wu J., Chen W. (2025). Bisphenol A Exposure and Neurodevelopmental Disorders
and Problems
in Children under 12 Years of Age: A Systematic Review and Meta-Analysis. Journal of Hazardous Materials.

[18] Persson L., Carney Almroth B. M., Collins C. D., Cornell S., de Wit C. A., Diamond M. L., Fantke P., Hassellöv M., MacLeod M., Ryberg M. W., Søgaard Jørgensen P., Villarrubia-Gómez P., Wang Z., Hauschild M. Z. (2022). Outside
the Safe Operating Space of the Planetary Boundary for Novel Entities. Environ. Sci. Technol..

[19] Knezevic E., Nenic K., Milanovic V., Knezevic N. N. (2023). The Role of Cortisol
in Chronic Stress, Neurodegenerative Diseases, and Psychological Disorders. Cells.

[20] Jones D. P. (2016). Sequencing
the Exposome: A Call to Action. Toxicol Rep.

[21] Lai Y., Koelmel J. P., Walker D. I., Price E. J., Papazian S., Manz K. E., Castilla-Fernández D., Bowden J. A., Nikiforov V., David A., Bessonneau V., Amer B., Seethapathy S., Hu X., Lin E. Z., Jbebli A., McNeil B. R., Barupal D., Cerasa M., Xie H., Kalia V., Nandakumar R., Singh R., Tian Z., Gao P., Zhao Y., Froment J., Rostkowski P., Dubey S., Coufalíková K., Seličová H., Hecht H., Liu S., Udhani H. H., Restituito S., Tchou-Wong K.-M., Lu K., Martin J. W., Warth B., Godri Pollitt K. J., Klánová J., Fiehn O., Metz T. O., Pennell K. D., Jones D. P., Miller G. W. (2024). High-Resolution
Mass Spectrometry for Human Exposomics: Expanding Chemical Space Coverage. Environ. Sci. Technol..

[22] Dennis K. K., Marder E., Balshaw D. M., Cui Y., Lynes M. A., Patti G. J., Rappaport S. M., Shaughnessy D. T., Vrijheid M., Barr D. B. (2017). Biomonitoring in
the Era of the Exposome. Environ. Health Perspect.

[23] Sdougkou K., Papazian S., Bonnefille B., Xie H. Y., Edfors F., Fagerberg L., Uhlén M., Bergström G., Martin L. J., Martin J. W. (2024). Longitudinal
Exposomics in a Multiomic
Wellness Cohort Reveals Distinctive and Dynamic Environmental Chemical
Mixtures in Blood. Environ. Sci. Technol..

[24] Jiang C., Wang X., Li X., Inlora J., Wang T., Liu Q., Snyder M. (2018). Dynamic Human Environmental Exposome Revealed by Longitudinal
Personal Monitoring. Cell.

[25] Hu X., Walker D. I., Liang Y., Smith M. R., Orr M. L., Juran B. D., Ma C., Uppal K., Koval M., Martin G. S., Neujahr D. C., Marsit C. J., Go Y. M., Pennell K. D., Miller G. W., Lazaridis K. N., Jones D. P. (2021). A Scalable Workflow to Characterize
the Human Exposome. Nat. Commun..

[26] Gu Y., Feuerstein M. L., Lloyd D. T., Patel C. J., Johnson C. H., Warth B. (2025). Quantitative
Exposomics Targeting over 200 Toxicants and Key Biomarkers
at the Picomolar Level. Environ. Sci. Technol..

[27] Chaker J., Kristensen D. M., Halldorsson T. I., Olsen S. F., Monfort C., Chevrier C., Jegou B., David A. (2022). Comprehensive Evaluation
of Blood Plasma and Serum Sample Preparations for HRMS-Based Chemical
Exposomics: Overlaps and Specificities. Anal.
Chem..

[28] Schymanski E. L., Kondic T., Neumann S., Thiessen P. A., Zhang J., Bolton E. E. (2021). Empowering Large Chemical Knowledge
Bases for Exposomics:
PubChemLite Meets MetFrag. J. Cheminf..

[29] Neumann S., Meier R., Wenk M., Elapavalore A., Nishioka T., Schulze T., Stravs M., Tsugawa H., Matsuda F., Schymanski E. L. (2026). MassBank:
An Open and FAIR Mass Spectral
Data Resource. Nucleic Acids Res..

[30] Peets P., Wang W. C., MacLeod M., Breitholtz M., Martin J. W., Kruve A. (2022). MS2Tox Machine Learning Tool for
Predicting the Ecotoxicity of Unidentified Chemicals in Water by Nontarget
LC-HRMS. Environ. Sci. Technol..

[31] Arturi K., Harris E. J., Gasser L., Escher B. I., Braun G., Bosshard R., Hollender J. (2025). MLinvitroTox
Reloaded for High-Throughput
Hazard-Based Prioritization of High-Resolution Mass Spectrometry Data. J. Cheminform.

[32] Lin E. Z., Esenther S., Mascelloni M., Irfan F., Godri Pollitt K. J. (2020). The Fresh
Air Wristband: A Wearable Air Pollutant Sampler. Environ. Sci. Technol. Lett..

[33] Hernandes V. V., Zeyda M., Wisgrill L., Warth B. (2025). Dried Blood Spots Analysis
for Targeted and Non-Targeted Exposomics. Environ.
Int..

[34] Thiele S., Martin J. W. (2025). A Critical Review
and Evaluation of Blood Microsampling
Devices for Exposomics. Environ. Sci. Technol.
Lett..

[35] Dark Metabolome Debate Continues: Siuzdak and Giera Respond. The Analytical Scientist. https://theanalyticalscientist.com/issues/2025/articles/june/the-dark-metabolome-debate-continues-siuzdak-and-giera-respond (accessed 2026-03-04).

[36] International
Human Genome Sequencing Consortium (2004). Finishing the Euchromatic Sequence of the Human Genome. Nature.

[37] Pennisi E. (2000). Genomics Comes
of Age. Science.

[38] Reuter J. A., Spacek D. V., Snyder M. P. (2015). High-Throughput
Sequencing Technologies. Mol. Cell.

[39] Cost of Sequencing a Human Genome. https://www.genome.gov/about-genomics/fact-sheets/Sequencing-Human-Genome-cost (accessed 2026-02-12).

[40] Masaryk University. European Infrastructure for human exposome (EIRENE). https://eirene.eu/ (accessed 2026-03-05).

[41] Network for Exposomics in the United States (NEXUS). https://www.nexus-exposomics.org/ (accessed 2026-03-05).

[42] David A., Lennon S., Mercier F., Bouhlel J., Chaker J., Appenzeller B. M. R., Audouze K., Ayeni K. I., Bailly-Chouriberry L., El Balkhi S., Barouki R., Belova L., Benmarhnia T., Bonnefille B., Bessonneau V., Bonvallot N., Bouchart V., Bouvier-Capely C., Buisson C., Bury D., Covaci A., Coumoul X., Debrauwer L., Delépée R., Denys S., Dereumeaux C., Feuerstein M. L., Fillol C., Gago-Ferrero P., Garcia P., Gicquel T., Gil-Solsona R., Grova N., Habchi B., Hernandes V. V., Iglesias-González A., Jamin E. L., Koch H. M., Lefèvre-Arbogast S., Lindh C., Louyer M.-V., Macheka L. R., Martin J. W., Marques M., Mol H., Ndaw S., Oleko A., Papazian S., Přibylová P., Price E. J., Schymanski E. L., Thiele S., Warth B., Zeman F., Blanc E., Antignac J.-P., Le Bizec B., Samson M. (2026). Mapping the Human Chemical Exposome for Public Health. Nat. Med..

[43] IHEN - The International Human Exposome Network. https://humanexposome.net/ (accessed 2026-02-14).

[44] Washington, D.C. Declaration on the Human Exposome. Exposome Moonshot Forum. https://exposomemoonshot.org/washington-d-c-declaration-on-the-human-exposome/ (accessed 2026-02-15).

[45] Vermeulen, R. Human exposome research: Potential, limitations and public policy implications | Think Tank | European Parliament. https://www.europarl.europa.eu/thinktank/en/document/EPRS_STU(2025)765791 (accessed 2026-02-18).

[46] Miller G.
W., Bennett L. M., Balshaw D., Barouki R., Bhutani G., Dolinoy D., Gao P., Jett D., Karagas M., Klánová J., Lein P., Li S., Metz T. O., Patel C. J., Pollitt K., Rajasekar A., Sillé F., Thessen A., Thuault-Restituito S., Vermeulen R., Ward-Caviness C. K., Wright R., Banbury Exposomics
Consortium (2025). Integrating Exposomics
into Biomedicine. Science.

